# Prenatal Fine Particulate Matter (PM2.5) Exposure and the Risk of Pediatric Inguinal Hernia or Hydrocele: A Retrospective Cohort Study †

**DOI:** 10.3390/jcm15083089

**Published:** 2026-04-17

**Authors:** Eun Jung Kim, Jin-Gon Bae, Eun-jung Koo

**Affiliations:** 1 Department of Urban Planning, Keimyung University, Daegu 42601, Republic of Korea; 2 Department of Obstetrics & Gynecology, Keimyung University School of Medicine, Daegu 42601, Republic of Korea; 3 Division of Pediatric Surgery, Department of Surgery, Keimyung University School of Medicine, Daegu 42601, Republic of Korea

**Keywords:** air pollution, fetal development, fine particulate matter, hydrocele, inguinal hernia, prenatal exposure

## Abstract

**Background/Objectives**: Inguinal hernia and hydrocele are common pediatric surgical conditions resulting from failed obliteration of the processus vaginalis during fetal development. Although prenatal exposure to fine particulate matter (PM2.5) has been linked to adverse perinatal outcomes and congenital anomalies, its role in structurally defined pediatric surgical diseases remains unclear. We examined the association between maternal PM2.5 exposure during pregnancy and the risk of inguinal hernia or hydrocele in offspring. **Methods**: We performed a retrospective cohort study of 1093 mother–offspring pairs delivering at a tertiary referral center (July 2016–June 2019). Monthly residential PM2.5 levels were estimated at geocoded maternal addresses using kriging interpolation from fixed-site monitoring stations. Offspring diagnosed with inguinal hernia or hydrocele through March 2024 were identified using ICD-10 codes. Perinatal characteristics were compared using *t*-tests and chi-square tests, and multivariable logistic regression assessed trimester-specific PM2.5 exposure and risk. **Results**: During follow-up, 53 offspring (4.85%) developed inguinal hernia or hydrocele. Male sex (odds ratio [OR], 24.71; 95% CI, 5.95–102.54; *p* < 0.001) and second-trimester PM2.5 exposure (OR, 1.07 per µg/m^3^; 95% CI, 1.01–1.14; *p* = 0.028) were independent risk factors. A dose–response pattern was observed across quartiles of second-trimester exposure; an interquartile range increase was associated with a 64% higher risk (OR, 1.64). The model showed good discrimination (AUC, 0.804). **Conclusions**: Elevated maternal PM2.5 exposure during the second trimester was independently associated with increased risk of inguinal hernia or hydrocele in offspring. Prenatal air pollution may contribute to persistence of the processus vaginalis and represents a potentially modifiable environmental risk factor.

## 1. Introduction

Inguinal hernia is a common indication for surgery in childhood. Reported incidence ranges from approximately 1–5% among term infants and increases substantially in premature populations [1,2,3,4,5]. Hydrocele is likewise prevalent in male infants, affecting approximately 0.5–6% of cases, with many resolving spontaneously during early infancy [6,7]. Despite differences in clinical course, both conditions share a common embryological origin: persistence of the processus vaginalis (PPV).

The processus vaginalis is a transient peritoneal evagination that accompanies testicular descent during fetal development [8]. Under normal physiological conditions, this structure undergoes obliteration after completion of descent. Failure of closure creates a patent peritoneal channel, permitting either herniation of abdominal contents or accumulation of peritoneal fluid, clinically manifesting as an inguinal hernia or hydrocele [9]. While the anatomical sequence leading to PPV has been extensively described, the determinants that modulate this developmental process remain insufficiently understood.

Inguinal hernia is not only common but also clinically significant due to the risk of incarceration, which may lead to bowel obstruction, ischemia, and the need for emergency surgical intervention [1,5]. Therefore, timely diagnosis and surgical management are essential for affected children.

Advances in minimally invasive surgical techniques have influenced the management of inguinal hernia and hydrocele. Laparoscopic approaches, including percutaneous internal ring suturing, have been increasingly adopted, offering advantages such as reduced operative trauma, improved cosmetic outcomes, and facilitating the evaluation of contralateral PPV [10]. These evolving surgical strategies highlight the clinical importance of understanding modifiable risk factors that may contribute to the development of these conditions.

Fine particulate matter with an aerodynamic diameter ≤2.5 μm (PM2.5) constitutes a major component of ambient air pollution. Owing to their small size, these particles can deposit in distal airways, enter systemic circulation, and affect multiple organs [11,12]. Increasing epidemiological evidence has linked maternal exposure to PM2.5 with impaired fetal growth, placental dysfunction, and structural congenital anomalies [13,14,15]. We have previously reported associations between prenatal PM2.5 exposure and congenital anomalies from a surgical perspective [16]. However, whether maternal exposure influences embryological processes governing peritoneal closure has not yet been investigated.

Given the hormonally regulated and temporally sensitive nature of testicular descent and peritoneal remodeling, environmental exposures during pregnancy may plausibly alter PPV regression. Therefore, in this study, we aimed to evaluate the relationship between trimester-specific maternal PM2.5 exposure and the subsequent diagnosis of inguinal hernia or hydrocele in offspring.

## 2. Materials and Methods

### 2.1. Study Design and Population

This retrospective birth cohort study included mother–offspring pairs delivering at a university-affiliated tertiary referral center between July 2016 and June 2019. Eligible participants were singleton live births to mothers residing in Daegu Metropolitan City during pregnancy. Multiple gestations, stillbirths, postnatal transfers from outside institutions, and cases lacking complete residential data were excluded.

To minimize confounding from established risk factors for inguinal hernia, the cohort was restricted to term infants (gestational age ≥ 37 weeks) with a birth weight ≥2500 g. Offspring with chromosomal abnormalities or major congenital malformations were excluded. Follow-up for outcome ascertainment was conducted through March 2024 using institutional electronic medical records.

### 2.2. Exposure Assessment

**Figure 1:**
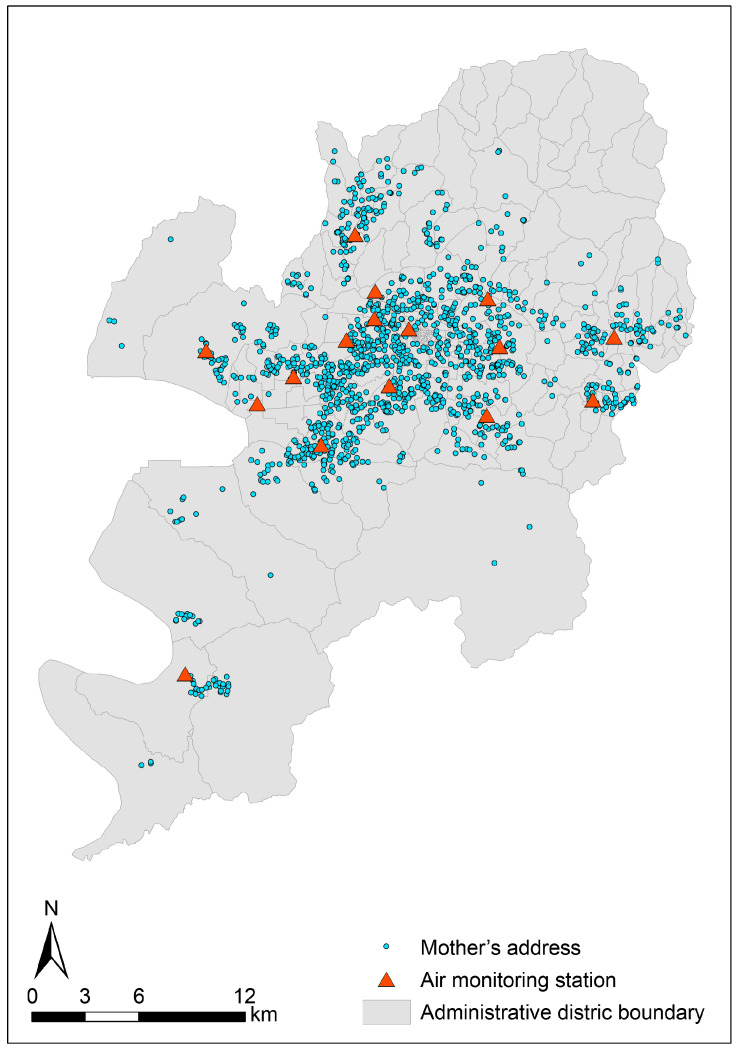
Location of air monitoring stations (N = 16) and maternal residences (N = 1093) in Daegu.

Ambient PM2.5 data were obtained from the national air quality surveillance network (AirKorea, Korea Environment Corporation, Seoul, Republic of Korea), which compiles measurements from monitoring stations across South Korea. We extracted PM2.5 measurements from 16 fixed-site monitoring stations operating in Daegu during the study period. Maternal residential addresses documented at delivery were geocoded, and the spatial distribution of maternal residences and monitoring stations within Daegu administrative districts is shown in Figure 1.

**Figure 2:**
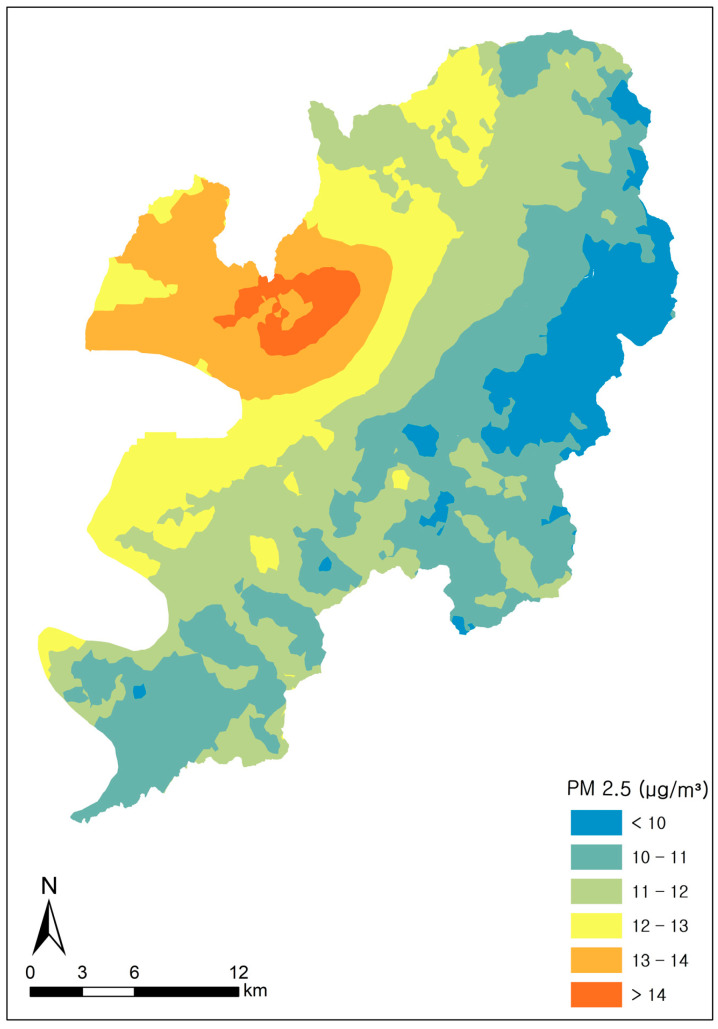
Predicted Mean PM2.5 (μg/m^3^) in Daegu, September 2018.

Rather than assigning exposure based solely on the nearest monitoring station (proximity-based assignment), we estimated PM2.5 concentrations at each residential location using a surface-based geostatistical approach. Spatial interpolation was performed using ArcGIS Pro version 3.2 (Esri Inc., Redlands, CA, USA) with the Geostatistical Wizard. Monthly prediction surfaces were generated using ordinary co-kriging, a variogram-based stochastic method that exploits spatial autocorrelation in PM2.5 and incorporates an auxiliary variable to improve spatial prediction. PM2.5 was modeled as the primary variable, and elevation (derived from topographic data) was included as a secondary variable. The output surface type was set to prediction, and a fixed search neighborhood of 15 neighbors was specified. Prediction surfaces were generated at a spatial resolution of 400 m (cell size = 400 m) and restricted to the Daegu administrative boundary by setting the processing extent and clipping the output surface to the city boundary. As an illustrative example of the monthly ordinary co-kriging prediction surfaces, Figure 2 presents the predicted mean PM2.5 concentration (µg/m^3^) in Daegu for September 2018.

Predicted PM2.5 concentrations were extracted at each geocoded residential location and used to derive pregnancy-period exposure metrics. Compared with proximity-based assignment, this approach may reduce exposure misclassification by incorporating information from multiple monitoring stations and better capturing within-city spatial gradients [17,18,19]. Spatial interpolation methods, including kriging and co-kriging, are widely used in environmental exposure assessment and in environmental and urban planning research to characterize the spatial distribution of particulate matter (PM10 and PM2.5) [20,21,22]. Monthly exposure estimates were aggregated to calculate trimester-specific mean PM2.5 concentrations for each pregnancy.

### 2.3. Outcome Definition

Offspring were categorized according to the presence or absence of an inguinal hernia or hydrocele diagnosed before March 2024. Diagnoses were initially identified using International Classification of Diseases, 10th Revision codes and subsequently confirmed through detailed medical chart review to ensure clinical validity:

- Inguinal hernia: K40.00–K40.90
- Hydrocele: N43.00–N43.30, P83.5

### 2.4. Covariates and Statistical Analysis

Covariates were selected based on clinical relevance and prior literature. To minimize overfitting, we considered the events-per-variable (EPV) ratio during model construction. Collinearity among variables was assessed using variance inflation factors, and no significant multicollinearity was identified.

Maternal variables included age, gestational duration, diabetes mellitus, hypertension, and smoking history. Neonatal variables included sex, gestational age, birth weight, Apgar scores at 1 and 5 min, and the presence of congenital anomalies.

All statistical analyses were performed using IBM SPSS Statistics version 29.0 (IBM Corp., Armonk, NY, USA). Normality of continuous variables was assessed using the Shapiro–Wilk test. Continuous variables are reported as means ± standard deviations and were compared using independent *t*-tests. Categorical variables were analyzed using chi-square or Fisher’s exact tests as appropriate. Missing data were minimal and handled using complete-case analysis.

Multivariable logistic regression was used to estimate adjusted odds ratios (ORs) with 95% confidence intervals (CIs) for associations between trimester-specific PM2.5 exposure and inguinal hernia or hydrocele. Model discrimination was assessed using the area under the receiver operating characteristic curve (AUC). Statistical significance was defined as a two-sided *p* < 0.05.

Model calibration was evaluated using the Hosmer–Lemeshow goodness-of-fit test and calibration plots. PM2.5 exposure during the second trimester was categorized into quartiles based on the empirical distribution of the study population, ensuring an equal number of participants in each group.

### 2.5. Ethics Statement

All data were de-identified prior to analysis. The study protocol was approved by the Institutional Review Board of Keimyung University Dongsan Medical Center (Approval No. 2025-04-061, approved on 8 May 2025) and conducted in accordance with the Declaration of Helsinki and applicable local regulations. The requirement for informed consent was waived due to the retrospective design and use of anonymized data.

## 3. Results

**Figure 3:**
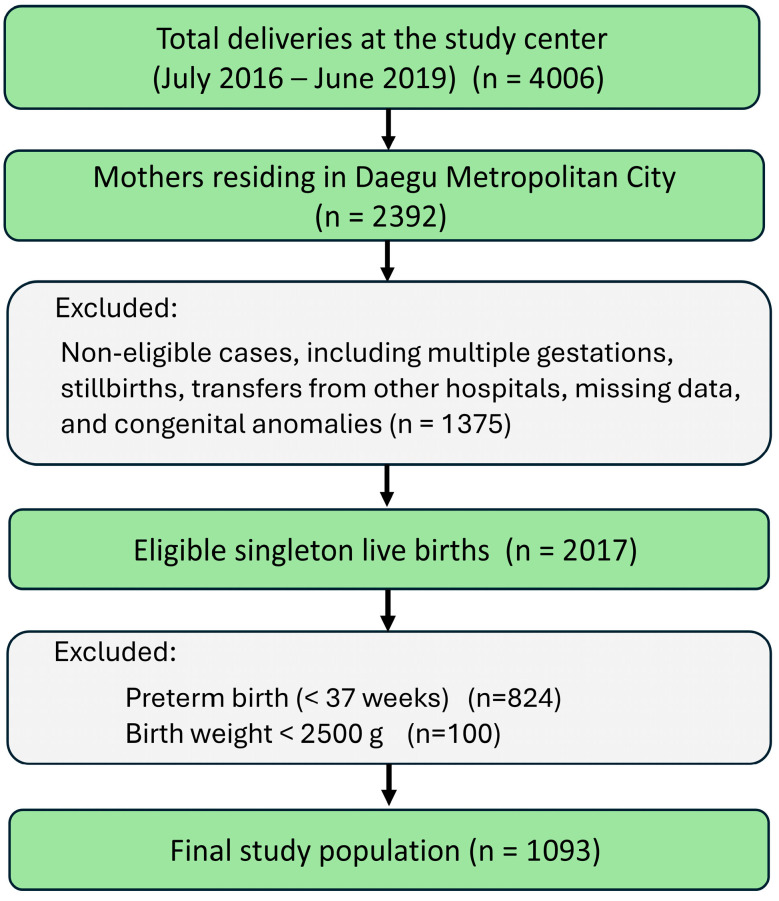
Flow diagram of study population selection.

The study population selection process is illustrated in Figure 3. A total of 1093 mother–offspring pairs were included in the analysis. The mean maternal age was 34.1 ± 4.5 years, and the mean gestational age at delivery was 38.6 ± 1.0 weeks. Among the offspring, 574 (52.5%) were male, and 519 (47.5%) were female. The mean birth weight was 3181 ± 377 g. The mean Apgar scores at 1 and 5 min were 7.8 ± 0.5 and 8.9 ± 0.3, respectively. Maternal comorbidities included diabetes mellitus (118 mothers, 10.8%), hypertension (75 mothers, 6.9%), and a history of smoking (six mothers, 0.5%) (Table 1).

Among the offspring, 53 (4.9%) were diagnosed with either inguinal hernia or hydrocele during follow-up. Comparison between affected and unaffected groups showed a significantly higher proportion of male offspring in the affected group (96.2% vs. 50.3%, *p* < 0.001). Although birth weight was slightly higher in affected offspring, the absolute difference was small and not independently associated with the outcome in multivariable analysis. No significant differences were observed between the groups in gestational age, Apgar scores, maternal age, or maternal comorbidities (Table 2).

Analysis of PM2.5 exposure by trimester showed that second-trimester exposure was significantly higher in the affected group than in the unaffected group (25.4 ± 4.6 µg/m^3^ vs. 23.9 ± 4.9 µg/m^3^, *p* = 0.032). No significant differences were observed in the first- or third-trimester exposure between the two groups (Table 3).

**Figure 4:**
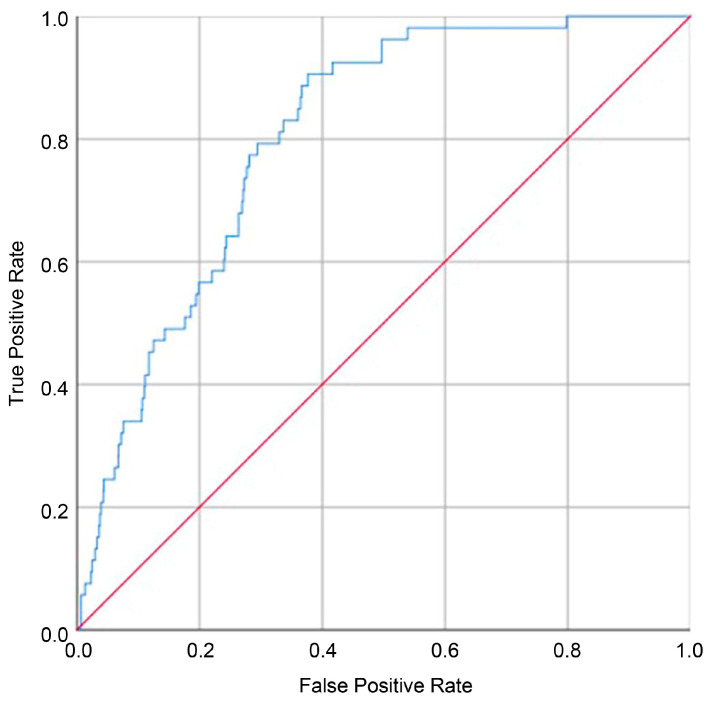
Receiver operating characteristic (ROC) curve for the multivariable model evaluating the risk of inguinal hernia or hydrocele. Area under the curve (AUC), 0.804. The blue line represents the ROC curve of the model, and the red line indicates the reference line corresponding to random classification (AUC = 0.5).

Multivariate logistic regression analysis was performed to identify independent risk factors for the development of inguinal hernia or hydrocele (Table 4). Male sex was strongly associated with the condition (OR, 24.71; 95% CI, 5.95–102.54; *p* < 0.001). Exposure to PM2.5 during the second trimester was a significant risk factor. Each 1-µg/m^3^ increase in second-trimester PM2.5 exposure was associated with a 7% increase in odds (adjusted OR, 1.07; 95% CI, 1.01–1.14). Other variables, including birth weight, maternal age, gestational age, maternal diabetes mellitus, maternal hypertension, and smoking history, were not statistically significant. The multivariable model demonstrated good discriminative performance (AUC, 0.804), suggesting acceptable internal predictive validity (Figure 4).

To quantify the effect size, we examined the interquartile range (IQR) increase in the second-trimester PM2.5 exposure. An increase across the IQR was associated with a 64% higher risk of inguinal hernia or hydrocele (OR, 1.64), indicating a clinically meaningful gradient of risk across typical exposure levels.

**Figure 5:**
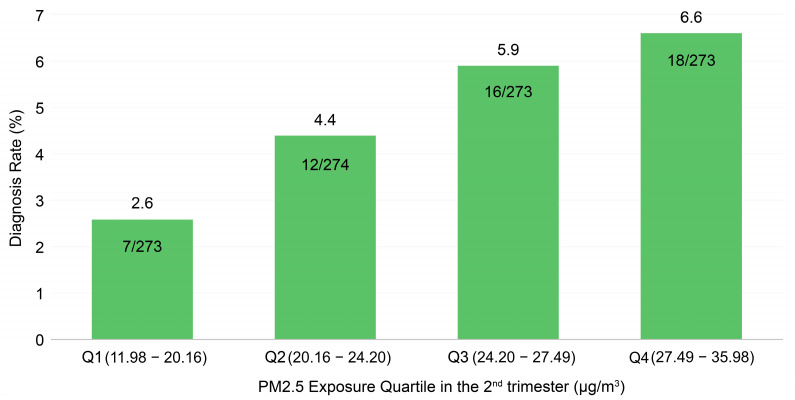
Incidence of inguinal hernia or hydrocele by quartiles of second-trimester PM2.5 exposure.

In addition, second-trimester PM2.5 concentrations were categorized into quartiles. A dose–response trend was observed, with incidence increasing from 2.6% in the lowest quartile to 6.6% in the highest quartile (as shown in Figure 5). This categorization showed a graded increase in incidence, supporting a dose–response relationship.

## 4. Discussion

In this cohort of 1093 mother–offspring pairs, maternal exposure to elevated PM2.5 concentrations during the second trimester was independently associated with increased odds of inguinal hernia or hydrocele in offspring. The association persisted after adjustment for relevant perinatal factors and demonstrated a graded exposure–response relationship, supporting the possibility of a sensitive mid-gestational window influencing PPV persistence.

Although the absolute difference in PM2.5 exposure between groups was modest and distributions overlapped, similar magnitudes of exposure differences have been associated with meaningful health outcomes in prior environmental epidemiological studies [13,14]. Given the relatively high baseline incidence of inguinal hernia in children, even modest increases in risk may translate into a meaningful population-level impact.

Both inguinal hernia and hydrocele originate from incomplete obliteration of the processus vaginalis [8,23,24]. The inguinoscrotal phase of testicular descent—occurring approximately between gestational weeks 25 and 35—is governed by androgen-mediated signaling, gubernacular differentiation, and coordinated extracellular matrix remodeling [23,24,25,26]. Disruption of these developmental mechanisms may prevent normal peritoneal regression, resulting in a persistent channel that predisposes individuals to postnatal pathology.

Airborne particulate pollutants have been shown to induce systemic oxidative stress, inflammatory activation, and endocrine perturbations during pregnancy [27,28,29]. Experimental and human studies suggest that prenatal exposure to environmental toxicants can impair fetal steroidogenesis and androgen-dependent pathways [30,31,32]. Given that androgen signaling plays a central role in gubernacular development and the timing of peritoneal closure, interference during critical developmental windows may plausibly alter PPV regression.

Previous epidemiologic studies have primarily examined prenatal air pollution in relation to fetal growth restriction, congenital anomalies, or cryptorchidism [13,14,15,16,33,34,35]. However, most investigations have focused on endocrine outcomes or testicular position rather than structural peritoneal persistence. By evaluating clinically confirmed inguinal hernia and hydrocele—conditions directly attributable to PPV—our findings extend environmental epidemiology into the domain of pediatric surgical outcomes.

Male sex was a strong independent predictor, consistent with established anatomical and developmental predispositions to PPV-related disorders. The marked predominance of male offspring in the affected group is consistent with known epidemiology. However, the observed ratio was higher than typically reported, which may be partly explained by the relatively limited follow-up period, as inguinal hernia in females may present later in childhood. Although inguinal hernia is less common in females, it may arise through similar embryological mechanisms involving the canal of Nuck [4]. Differences in timing and clinical presentation may contribute to sex-specific incidence patterns.

Preterm birth and low birth weight are recognized risk factors for inguinal hernia [36]; to minimize confounding, we restricted the cohort to term infants with a birth weight ≥ 2500 g and excluded major congenital anomalies. Although birth weight differed slightly in univariate analysis, it was not independently associated with the outcome in multivariable models, suggesting limited clinical relevance.

The trimester-specific association observed in this study suggests differential susceptibility across gestation. Although final testicular descent occurs later in pregnancy, molecular priming and mesenchymal remodeling begin earlier. Environmental perturbations during mid-gestation may therefore exert downstream anatomical consequences.

The exposure–response gradient further strengthens the interpretability of the finding. The quartile-based categorization resulted in unequal exposure ranges due to the empirical distribution of PM2.5 concentrations. However, sensitivity analyses using alternative categorization approaches yielded consistent trends, supporting the robustness of the observed dose–response relationship. While the per-unit effect size was modest, the relatively high baseline incidence of pediatric inguinal hernia implies that even a moderate increase in risk could translate into a meaningful population-level impact.

Several limitations warrant consideration. This study was conducted at a single tertiary referral center, which may limit the generalizability of the findings; therefore, caution is warranted when extrapolating these results to broader populations. Exposure estimates were derived from residential locations and did not incorporate indoor pollution, occupational exposures, or maternal mobility patterns. Such misclassification is likely non-differential and may bias the results toward underestimation of the true effects. In addition, important potential confounders, including socioeconomic status, co-exposure to other air pollutants, and lifestyle factors such as physical activity and maternal obesity, were not available and could not be adjusted for in this analysis. Given the observational nature of this study, causal inference is limited, and the findings should be interpreted as associations rather than evidence of causation. The relatively small number of outcome events (n = 53) resulted in an EPV ratio of approximately 7.6, which is slightly below the commonly recommended threshold and may raise concerns regarding model stability. However, sensitivity analyses using reduced models yielded consistent results, supporting the robustness of the findings.

Nonetheless, this study has several strengths, including a well-characterized birth cohort with extended follow-up, trimester-specific exposure modeling using geospatial methods, clinically validated outcomes, and multivariable adjustment for key confounders. These findings suggest that prenatal air pollution may influence the structural developmental processes underlying pediatric surgical disease.

## 5. Conclusions

Maternal exposure to higher levels of PM2.5 during mid-gestation was associated with an increased risk of inguinal hernia or hydrocele in offspring. Although causality cannot be established in this observational study, the trimester-specific and dose–response patterns observed support the hypothesis that ambient air pollution may contribute to the PPV. Strategies aimed at reducing prenatal exposure to fine particulate matter may have implications not only for cardiopulmonary and obstetric outcomes, but also for pediatric surgical morbidity. Prospective multicenter studies incorporating individualized exposure assessment and mechanistic biomarkers are warranted.

## Figures and Tables

**Table 1:** Demographic characteristics of the study population.

Study Population	Characteristic	Values
Mother (n = 1093)	Age (years)	34.1 ± 4.5 (18–46)
	Diabetes mellitus, n (%)	118 (10.8)
	Hypertension, n (%)	75 (6.9)
	Smoking history, n (%)	6 (0.5)
Offspring (n = 1093)	Gestational age (weeks)	38.6 ± 1.0 (37–42)
	Sex, male:female, n (%)	574:519 (52.5:47.5)
	Birth weight (g)	3181 ± 377 (2060–4610)
	Apgar score (1 min)	7.8 ± 0.5
	Apgar score (5 min)	8.9 ± 0.3

Values are presented as means ± standard deviations (SD; ranges) or numbers (%).

**Table 2:** Comparison of offspring characteristics between those with and without inguinal hernia or hydrocele.

Variable	No Hernia/Hydrocele (N = 1040)	Hernia/Hydrocele (N = 53)	*p*-Value
Sex, male:female, n (%)	523:517 (50.3:49.7)	51:2 (96.2:3.8)	<0.001
Gestational age (weeks)	38.6 ± 1.0	38.5 ± 0.9	0.352
Birth weight (g)	3176 ± 377	3296 ± 375	0.023
Apgar score at 1 min	7.8 ± 0.5	7.8 ± 0.4	0.362
Apgar score at 5 min	8.9 ± 0.3	8.9 ± 0.3	0.857
Maternal age (years)	34.0 ± 4.5	34.8 ± 4.4	0.222
Maternal diabetes mellitus, n (%)	112 (10.8)	6 (11.3)	0.822
Maternal hypertension, n (%)	73 (7.0)	2 (3.8)	0.518
Maternal smoking, n (%)	6 (0.6)	0 (0.0)	1.000

Values are presented as numbers (%) or means ± standard deviations (SD). *p*-values were calculated using independent *t*-tests for continuous variables and chi-square tests or Fisher’s exact tests for categorical variables.

**Table 3:** Comparison of PM2.5 exposure between offspring with and without inguinal hernia or hydrocele by trimester.

Trimester	No Hernia/Hydrocele (mean ± SD)	Hernia/Hydrocele (mean ± SD)	*p*-Value
1st trimester	24.9 ± 4.3	24.0 ± 4.9	0.151
2nd trimester	23.9 ± 4.9	25.4 ± 4.6	0.032
3rd trimester	24.0 ± 5.3	24.9 ± 5.0	0.265

Values are presented as means ± standard deviations (SD). *p*-values were calculated using independent *t*-tests.

**Table 4:** Multivariate logistic regression analysis of factors associated with inguinal hernia or hydrocele.

Variable	Odds Ratio (OR)	95% CI	*p*-Value
Sex of offspring (male)	24.71	5.95–102.54	<0.001
Birth weight (g)	1.001	1.000–1.001	0.121
PM2.5 in 2nd trimester (µg/m^3^)	1.070	1.01–1.14	0.028
Gestational age (weeks)	0.783	0.57–1.09	0.143
Maternal age (years)	1.036	0.97–1.11	0.301
Maternal diabetes mellitus	0.781	0.31–1.96	0.599
Maternal hypertension	2.389	0.55–10.37	0.245

Values are presented as odds ratios (OR) with 95% confidence intervals (CI). *p* < 0.05 was considered statistically significant.

## Data Availability

The datasets analyzed during the current study are not publicly available due to institutional regulations, but are available from the corresponding author upon reasonable request and approval from the Institutional Review Board.

## Abbreviations

AUC	Area Under the Curve
ICD-10	International Classification of Diseases, 10th Revision
IQR	Interquartile Range
IRB	Institutional Review Board
PM2.5	Fine particulate matter ≤ 2.5 μm in diameter
PPV	Persistent Processus Vaginalis
ROC	Receiver Operating Characteristic
